# Improved Monitoring of Wildlife Invasion through Data Augmentation by Extract–Append of a Segmented Entity

**DOI:** 10.3390/s22197383

**Published:** 2022-09-28

**Authors:** Jaekwang Lee, Kangmin Lim, Jeongho Cho

**Affiliations:** Department of Electrical Engineering, Soonchunhyang University, Asan 31538, Korea

**Keywords:** object detection, surveillance, semantic segmentation, data augmentation

## Abstract

Owing to the continuous increase in the damage to farms due to wild animals’ destruction of crops in South Korea, various methods have been proposed to resolve these issues, such as installing electric fences and using warning lamps or ultrasonic waves. Recently, new methods have been attempted by applying deep learning-based object-detection techniques to a robot. However, for effective training of a deep learning-based object-detection model, overfitting or biased training should be avoided; furthermore, a huge number of datasets are required. In particular, establishing a training dataset for specific wild animals requires considerable time and labor. Therefore, this study proposes an Extract–Append data augmentation method where specific objects are extracted from a limited number of images via semantic segmentation and corresponding objects are appended to numerous arbitrary background images. Thus, the study aimed to improve the model’s detection performance by generating a rich dataset on wild animals with various background images, particularly images of water deer and wild boar, which are currently causing the most problematic social issues. The comparison between the object detector trained using the proposed Extract–Append technique and that trained using the existing data augmentation techniques showed that the mean Average Precision (mAP) improved by ≥2.2%. Moreover, further improvement in detection performance of the deep learning-based object-detection model can be expected as the proposed technique can solve the issue of the lack of specific data that are difficult to obtain.

## 1. Introduction

**Figure 1:**
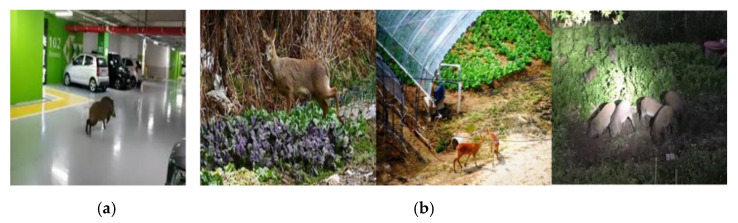
Examples of the threats and damage by wild animals: (**a**) appearance of wild animals in urban areas; (**b**) destruction of crops by wild animals.

Damage to crops due to attacks by wild animals is one of the primary reasons for a reduction in crop yield. As with indiscriminate logging and the expansion of urban environments, including roads and buildings, incidents of crop attacks by wild animals have increased as they have lost their habitats. According to the Ministry of Environment in South Korea, the amount of damage to crops by wild animals between 2014 and 2018 was ~57 billion KRW, which is 11.4 billion KRW annually; the damage by wild boars and water deer is the largest [1]. Water deer are listed as endangered in the International Union for Conservation of Nature Red List of Threatened Species. Wild boars usually inhabit deep mountains and areas with broad-leaved trees, but during the mating season or preparation for winter, they often come down to urban areas in search of food. Particularly, the ecosystems near urban areas do not have the predators of wild boar; therefore, their population increases. There are several incidents where water deer and wild boars, having had a huge increase in population, destroy crops and appear in residential areas, causing damage to people’s life or properties [1] (Figure 1).

To mitigate such damages, farms have attempted to dispel animals by installing electrical fences or using sound and light via warning lamps and explosive ultrasound. However, electrical fences may lead to casualties and, if damaged, may cause high maintenance costs. Additionally, warning lamps or explosive ultrasound can become less effective in the future as animals get accustomed to them. Recently, methods that prevent the invasion of wild animals have been proposed, which use robots equipped with deep learning-based object-detection technology to detect wild animals and use LEDs and alarms only when they detect objects in real-time monitoring [2]. However, deep learning-based object-detection technology requires sufficient data to train the deep learning model. Currently, training data are collected either by directly taking pictures of objects, extracting images from video recordings, or web crawling. However, there are certain limitations humans have in acquiring images of wild animals, such as access challenges, leading to challenges in model training. Overfitting can also be an issue while training a model in such a case [3,4,5,6,7].

To overcome the aforementioned data-collection issues, a large amount of training data can be generated via data augmentation [8,9]. Data augmentation is a technique that increases the amount of limited data artificially by increasing the number of images through applying different types of transformation to an original image. Although various data augmentation techniques have been proposed, there are still many limitations in performing data augmentation with a limited amount of data. Therefore, this study proposes an Extract–Append data augmentation method where only the objects of interest, specifically wild boars and water deer, are extracted from a minimal number of images via semantic segmentation, and corresponding objects are appended automatically to numerous background images. Masks, the shapes of the objects to be extracted from the segmentation network, are acquired, and the segmented objects are produced by the binarization and synthesis process. Later, the augmented training data are acquired from the inverse binarization and the synthesis of various background images. This study compared and evaluated the object-detection performance of the proposed and existing data augmentation methods to verify the usefulness of the proposed method. The contribution of this study is as follows:

1. It proposes the Extract–Append data augmentation method, which automatically generates a large amount of diverse data by extracting the masks of the objects of interest from segmentation and synthesizing them with countless arbitrary backgrounds.
2. It enables the synthesis of the object with various backgrounds without losing the original object shape by suggesting a data-processing method, which synthesizes the extracted object with the background image after creating a space so that the extracted object shape can be maintained as accurately as possible on the arbitrary background image.
3. It provides a method that could extract the mask of an object to facilitate additional training automatically, even if new background images were acquired later based on the previously trained model on a specific object.

The rest of the article comprises the following sections: in Section 2, the related research on data augmentation is described; in Section 3, the proposed Extract–Append data augmentation technique is explained; in Section 4, the test process and results are presented; and, finally, in Section 5, the present study is concluded.

## 2. Related Works

Generally, data augmentation uses spatial-level transformation and pixel-level transformation. The former involves applying spatial changes to an object. For example, it includes flipping, rotating, and cropping [10,11,12,13]. In contrast, the latter involves pixel-level image transformation and includes contrast, which adjusts the ratio of contrast in an image, and the addition of random noise to increase the adaptability of the data under various environments [14,15]. Other methods have been proposed, including cutout, which removes a part of the image by randomly masking it with squares [16], or mixup [17], which generates new data by mixing up two images by a certain ratio. However, if data augmentation is performed with a minimum number of images, only the images with limited backgrounds (environments) are produced, which makes it difficult to expect an improvement in the performance of the detection model, and in this case, data augmentation via cutout or mixup can instead play the role of noise [18,19].

Various data augmentation methods have been proposed to resolve these issues. D. Yorioka et al. [20] attempted to solve the lack of data by generating a significant number of fake images based on GAN; however, GAN training requires tremendous time, and it is difficult to train a GAN model effectively with a minimal number of data. V. Olsson et al. [21] proposed ClassMix, which increased the amount of data by synthesizing the backgrounds and objects extracted from the segmentation. However, this requires training of objects and environments, and in synthesizing the extracted objects and environments, some information can be lost. S. Bang et al. [22] proposed a method to extract the objects in an image by masking and generating backgrounds from the masked space via GAN; however, under the condition where only a limited number of data could be used, the GAN-based background-generation process may result in distorted backgrounds and a long training time. G. Ghiasi et al. [23] suggested a method that arbitrarily selects two images and, after random scaling, attaches the object to another image. However, even this method cannot overcome the issue of diversity if a small number of images limits it, and it cannot avoid the degradation of image resolution during the random scaling process. Table 1 summarizes the strengths and weaknesses of existing and proposed augmentation techniques.

## 3. Methodology

**Figure 2:**
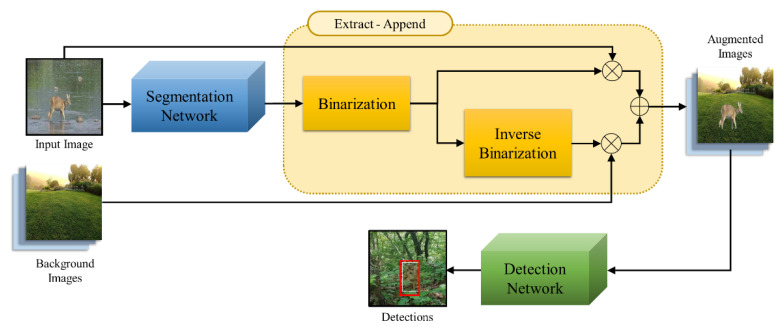
Block diagram of the object-detection system, including the proposed Extract–Append technique.

The existing data augmentation techniques can enable augmentation only for acquired image data. Therefore, they are limited in diversity and in the number of images that can be augmented. This study proposes the Extract–Append technique that can generate a large amount of diverse data by extracting objects using masks obtained through segmentation and synthesizing them with arbitrary backgrounds to solve these problems. The acquired limited image produces the mask of an object through a segmentation network. Subsequently, the binarization process transforms it into a binary mask, which is synthesized with an input image to extract the concerned object. The binary mask is again transformed into a mask to secure a space in the object’s shape, which is to be added to an arbitrary background through the inverse binarization process. Synthesis of the transformed mask and the new background image produces a background image with an object-shaped space, and then it is added to the extracted object to create a new image. The augmented image data are used in training the detection network. Figure 2 gives an overview of the object-detection system, including the proposed Extract–Append technique.

### 3.1. Semantic Segmentation

One of the most important application areas in image processing is segmentation, which categorizes and classifies images into similar regions in terms of semantic or cognitive perspectives. Here, semantic segmentation is a technique that can discern objects not by simple boundaries but by semantic regions and aims to classify objects by determining what each object signifies in an image that contains various objects, including cars, people, animals, and trees. When classifying an object, all pixels are grouped and categorized according to similar colors; through this classification, the mask of an object is extracted. To extract a more accurate mask for an object, manual photoshopping or GrabCut could be used; however, the study considered semantic segmentation to automatically extract the mask of specific objects universally. Generally, semantic segmentation is in an encoder–decoder structure. The encoder gradually performs downsampling to reduce the amount of calculation based on the size of an input image and improve the calculation speed to extract and compress the features of the object information to be extracted. However, the decoder performs upsampling to recover the lost spatial information due to reducing the spatial dimension in the encoder, and gradually attempts to recover clear object boundaries. In this way, semantic segmentation extracts the mask containing the object’s information [24,25,26].

### 3.2. Extract–Append for Data Augmentation

The proposed Extract–Append data augmentation process is summarized in Algorithm 1, in which the shape of an object is extracted using the mask of the said extracted object once the segmentation network training is completed, and the object is appended to various arbitrary backgrounds.
**Algorithm 1** Extract–Append Algorithm**Require:** Pretrained semantic segmentation model Φ **Input:** Input image containing an object $I_{obj}$, Background image $I_{back}$ **Output:** Create new image $A_{obj}$ 1: $M_{S}\leftarrow$ $\Phi \left(I_{obj}\right)⊳$ Extract the mask of an object 2: $\hat{M_{S}}=\left\{\begin{array}{c} 1,\;object\;\\ 0,background \end{array}\right.\;\leftarrow$ Binarization of $M_{S}$ 3: **for** each iteration do 4: $E_{obj}\leftarrow \;I_{obj}\;⨀\;\hat{M_{S}}\;⊳\;$ Extract an object from $I_{obj}$ 5: $C_{back}\leftarrow \;I_{obj}\;⨀\;\left|1-\hat{M_{S}}\right|⊳\;$ Making room for object insertion in $I_{back}$ 6: $A_{obj}\;\leftarrow \;E_{obj}+C_{back}$ 7: **end for**

From the image $I_{obj}$, acquired from web crawling and video frames that include the concerned object, the RGB 3-channel mask $M_{S}$ of the object is extracted via the segmentation network Φ. This is transformed into a 1-channel binary mask $\hat{M_{S}}$ that has either a 0 or 1 value via the binarization process, and the object $E_{obj}$ extracted by using this mask can be derived as in the following equation:

(1) $$E_{obj}=I_{obj}\;⨀\;\hat{M_{S}}\;$$

Here, ⨀ refers to a dot product. In other words, since the object has a value of 1 in the binary mask, the dot product of the binary mask and the input image results in a black background, and only the object retains its original color. In this way, the object is solely extracted. Later, to synthesize the extracted object with an arbitrary background $I_{back}$, an inversion of the binary mask is again performed so that the background is 1 and the object is 0. The dot product of the transformed mask and an arbitrary background results in an arbitrary background $C_{back}$ that has a value of 0 in the space of the object’s shape to be appended, as shown in the following equation:

(2) $$C_{back}=I_{back}⨀\;\left|1-\hat{M_{S}}\right|$$

If $E_{obj}$ with only object information is added to this, a new image $A_{obj}$, which is an arbitrary background with the appended object, is created.

(3) $$A_{obj}=E_{obj}+C_{back}$$

**Figure 3:**
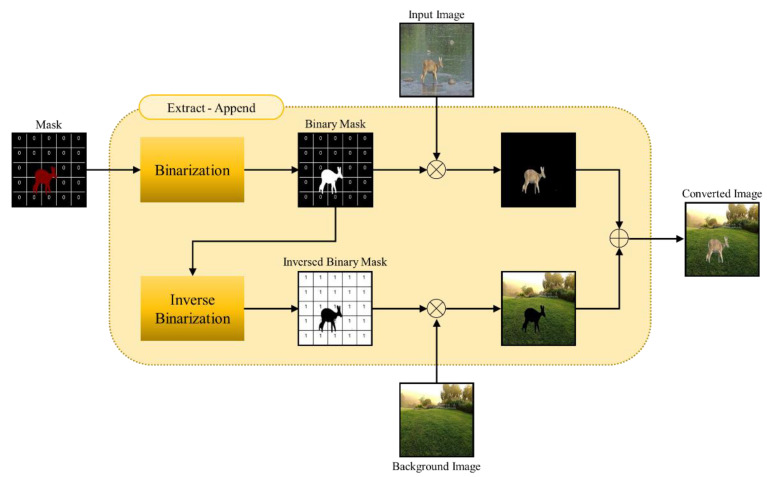
Block diagram of the proposed Extract–Append data augmentation process.

A detailed block diagram of the proposed Extract–Append data augmentation process is illustrated in Figure 3.

### 3.3. Object Detection

A large amount of augmented data generated by the proposed technique are used in the deep learning-based object-detection model for the surveillance of wild animals—the ultimate aim of this study. The detection model allowed for real-time processing and considered the You Only Look Once (YOLO) network, a one-stage detection method that performs classification and localization simultaneously. YOLO categorizes the input image into grids of S × S size, and each grid cell estimates B number of bounding boxes and the bounding box’s confidence score (CS). Here, a bounding box has five pieces of information (x, y, w, h, and C). x and y are the box’s central coordinates, corresponding to the boundary of the grid cell, and w and h also refer to the width and height, corresponding to the grid cell. Finally, C refers to the probability that the bounding box is included in a specific object. CS is the multiplication of the probability that the bounding box is included in the object $PR_{obj}$ and the Intersection over Union (IoU), the width of the overlapping region between the estimated and real values, and refers to the degree of confidence that an object exists within the bounding box as shown below.

(4) $$\;\mathrm{CS}\;=PR_{obj}\times \;\mathrm{IoU}$$

Each grid cell estimates the CS of N number of classes, and Conditional Class Probability (CCP)—which is the probability that if an object exists in a cell, it will be the k st class—is defined as shown below.

(5) $$\mathrm{CCP}\;=\mathrm{PR}\left(Class\left(k\right)|Object\right)$$

Therefore, the class-specific CS (CCS), which refers to how identical the probability that a specific object exists in each bounding box is to the actual value, can be summarized as below. The bounding box with the highest CCS among B number of bounding boxes that each grid cell estimated for an object is determined to be the bounding box for the said object [27].

(6) $$\mathrm{CSS}\;=PR_{obj}\times \;\mathrm{IoU}\;\times \;\mathrm{CCP}$$

## 4. Experimental Results

To realize a model for monitoring wild animals, such as water deer and wild boars, through the proposed data augmentation structure, the mask of objects should be first extracted, and to this end, the study used the segmentation model DeepLabv3+ [24]. The study trained the object-detection model using augmented data after embodying an Extract–Append processor based on the extracted mask of the object and attempted to validate the usefulness of the proposed method by evaluating the model’s detection performance. As a model for evaluating the object-detection performance, the study considered YOLOV4-tiny, and the training was performed with a NVIDIA RTX 3060 and Intel Core i7-1200F CPU. The reason for choosing YOLOV4-tiny among the various YOLO models is because it allows for an easy realization of an onboard embedded system and real-time processing. Its processing speed is relatively much faster than more recent models while its performance is slightly poor. A high-performance computer needs to be used to realize object detection using YOLOv4 in an actual farm, but this is unrealistic to carry out. In contrast, YOLOV4-tiny can allow for real-time object recognition on an embedded single board computer, such as Jetson Nano.

**Figure 4:**
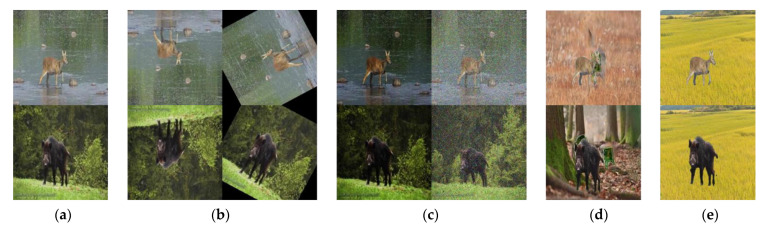
Example of water deer and wild boar images transformed by data augmentation: (**a**) original image (D1); (**b**) spatial-level transformation (D2); (**c**) pixel-level transformation (D3); (**d**) cut-and-paste (D4); (**e**) Extract–Append (D5).

The resolution of the input images was 416 × 416, and to compare the performance of the proposed Extract–Append technique with the existing data augmentation-based object-detection performance, the study categorized the dataset used in training into five types. Dataset D1 used in the evaluation test was created assuming that only 60 images per class were acquired by the image extraction from the videos or web crawling of wild boars and water deer, according to the aim of augmenting the data with a minimal number of images acquired limitedly. Dataset D2 was created with 480 images per class by adding the data transformed from D1 via spatial-level transformation. Augmentation by spatial-level transformation is one of the most widely used data augmentation techniques, and thus, it was included in all dataset constructions, except for D1. Dataset D3 was created with 540 images per class by image-contrast augmentation, one of the pixel-level transformation techniques. Cut-and-paste augmentation was used to create Dataset D4, with 1080 images per class, which is similar to the Extract–Append technique proposed in this study. Finally, Dataset D5 was created with 1480 images per class by Extract–Append augmentation, which conveniently allows for synthesizing objects with unlimited, arbitrary background images. The object-detection performance evaluation via the proposed data augmentation used 100 images per class. Figure 4 shows examples of the results from the data augmentation technique used in the training of the object-detection model.

The evaluation index for validating the performance of the model used the mean Average Precision (mAP), with Precision and Recall defined as follows:

(7) $$\mathrm{Precision}=\frac{\mathrm{TP}}{\mathrm{TP}+\mathrm{FP}}\;$$

(8) $$\mathrm{Recall}=\frac{\mathrm{TP}}{\mathrm{TP}+\mathrm{FN}}$$

Here, TP refers to True Positive, meaning that an object that needs to be detected was detected; FP refers to False Positive, meaning that an object that should not be detected was detected; FN refers to False Negative, meaning that an object that should not be detected was not detected; Precision, meaning accuracy, refers to the ratio of the objects detected by the model that was correctly detected; and Recall, meaning reproduction, refers to the ratio of the objects that should be detected and were correctly detected by the model. A PR curve is the cumulation of Precision and Recall from the highest CS, which is a value that expresses how accurately a model detects an object. The x-axis is Recall, and the y-axis is Precision. While the PR curve can determine the Precision value by the change in Recall, it is inconvenient to compare the performance of each technique quantitatively. To solve this inconvenience, the Average Precision, the area below the PR curve, is used, and the performance of each technique is evaluated by mAP, which is the Average Precision of each object if there are multiple objects divided by their numbers.

The study performed an evaluation test by changing IoU to examine the performance level of the object detector trained with the data generated by the proposed Extract–Append technique; the result is presented in Table 2. With IoU at 0.3, the performance of the object detector trained with the proposed D5 improved by 0.6% and 2.6% at minimum and maximum, respectively, based on mAP compared with that of the object detectors trained with D1 to D4. In addition, even if IoU increased to 0.5, the object detector trained with D5 showed a higher performance of 0.8% and 3.7% at minimum and maximum, respectively, than the object detectors trained with D1 to D4. Furthermore, when IoU is 0.7, the performance of the object detector based on the proposed technique improved by up to 34.8%, showing a 2.1% higher improvement from the object detector trained with D4, which is similar to the Extract–Append approach. It should be noted here that since D2 augments only a limited number of images, it is restricted in the number of images to be augmented as it faces the issue of the diversity of data. As discussed earlier, D3 can rather degrade the performance of an object detector as it adds noise to the limited number of images, thus showing the smallest performance improvement among all the data augmentation techniques used in the evaluation test. D4 extracts objects from existing images and fills the extracted space with RGB, similar to the background, using GAN. However, at this point, GAN takes a long time to be trained, and, as shown in Figure 4d, noise is added to the generated image, leading to limited performance improvement. Furthermore, it uses the existing background again; thus, as in D2 discussed above, it is limited with respect to the diversity of data. In contrast, the proposed D5 uses semantic segmentation to extract and synthesize an object to an arbitrary, intact background image. Therefore, the training time is much shorter than the method based on GAN, and since the synthesis uses various arbitrary background images, it can solve the data diversity issue. Furthermore, there is no limitation in the number of data to be augmented, resulting in a better performance of the object detector than the one trained with D1 to D4.

**Figure 5:**
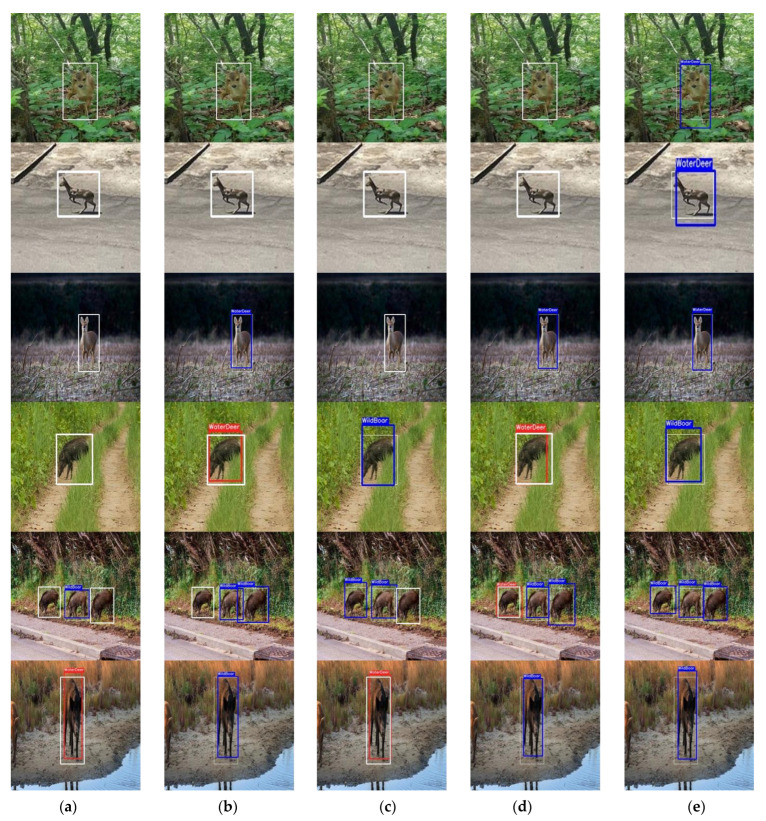
Example of water deer and wild boar detection results by data augmentation techniques: (**a**) data augmentation not applied (D1); (**b**) spatial-level transformation (D2); (**c**) pixel-level transformation (D3); (**d**) cut-and-paste (D4); (**e**) Extract–Append (D5).

Figure 5 illustrates the examples of the results of the object-detection model trained with data augmented by each technique, with the IoU at 0.7. The blue box in the figure is the result where the model identified the object correctly, the red color indicates that the model incorrectly identified the object as another object, and the white color shows the ground-truth.

## 5. Conclusions

An unbiased, diverse, and significant amount of data is necessary when training a deep learning-based object-detection model. Notably, building a training dataset for specific objects requires considerable time and labor, and generally, this is resolved through data augmentation. However, existing data augmentation techniques rely on spatial- or pixel-level transformation of images, which is limited in augmenting data based on a minimal number of images, resulting in a degraded model performance and the problem of diversity of training images. Therefore, this study proposed an Extract–Append data augmentation technique to resolve the issue of a lack of specific data and promote the performance improvement of a deep learning-based object-detection model. The proposed data augmentation technique extracts only specific objects through semantic segmentation, generates a diverse and vast amount of augmented training data from synthesis with varying arbitrary background images, and synthesizes the data without changing the shape of the extracted objects. The study conducted a performance comparison test between the object detector based on the proposed Extract–Append technique and the others based on the existing data augmentation techniques, which demonstrated that the object detector trained with the proposed approach showed a detection performance improvement of up to 34.8%. In addition, compared to the cut-and-paste technique, the proposed technique improved the detection performance by 2.1%. Following these results, it is anticipated that the proposed data augmentation technique can solve the issues of lack of data and diversity to enhance the performance of various deep learning-based rare object-detection models. In the future, we will conduct additional training on other rare objects besides water deer and wild boars. We aim to generalize the proposed data augmentation technique by synthesizing these objects with various background images. Furthermore, we will also continue to complement the mask-extraction technique, which lacks data in the segmentation process.

## Figures and Tables

**Table 1:** Comparison of the strengths and weaknesses of existing and proposed augmentation techniques.

Augmentation Method	Strengths	Weaknesses
Conventional	- We can create additional images by changing the direction and angle of the object based on the acquired image. - We can obtain additional images by adjusting the contrast ratio of the acquired image or adding noise to the image.	- Because it is augmented using only the collected images, there is a limit to the diversity of the object’s environment. - Regardless of the object, every pixel within the image may be transformed, changing the object’s unique characteristics.
Proposed	- Objects in the collected image can be combined with various random backgrounds to create an unlimited variety of data. - The mask for the object is extracted through segmentation and combined with a random background, so it is very unlikely to act as noise.	- The object’s mask quality is determined by its segmentation performance. - There is a slight sense of heterogeneity because the object is pasted on a random background after extraction.

**Table 2:** Comparison of the object-detection performance by data augmentation techniques.

Data	Data Augmentation				Class	AP0.3	AP0.5	AP0.7	mAP0.3	mAP0.5	mAP0.7
	Spatial-Lev. Trans.	Pixel-Lev. Trans.	Cut- Paste	Extract- Append							
D1					WaterDeer WildBoar	93.0 93.9	91.7 93.0	51.3 63.8	93.9	92.4	57.8
D2	O				WaterDeer WildBoar	95.8 93.7	95.5 93.7	89.4 88.7	94.8	94.4	89.0
D3	O	O			WaterDeer WildBoar	93.6 93.5	93.4 92.6	65.1 71.6	93.5	93.0	68.3
D4	O		O		WaterDeer WildBoar	97.0 94.8	96.9 93.7	92.1 88.9	95.5	95.3	90.5
D5	O			O	WaterDeer WildBoar	97.2 95.1	97.2 95.1	94.3 91.0	96.1	96.1	92.6

## Data Availability

The data presented in this study are available on request from the corresponding author.
